# Effects of Increased Nitrogen Availability on C and N Cycles in Tropical Forests: A Meta-Analysis

**DOI:** 10.1371/journal.pone.0144253

**Published:** 2015-12-03

**Authors:** Marylin Bejarano-Castillo, Julio Campo, Lilia L. Roa-Fuentes

**Affiliations:** 1 Pronatura Sur, A.C. Pedro Moreno 1, 29250, San Cristóbal de Las Casas, Mexico; 2 Instituto de Ecología, Universidad Nacional Autónoma de México, México D.F., Mexico; 3 Centro del Cambio Global y la Sustentabilidad en el Sureste, A.C., Villahermosa, Mexico; Institution and Department: Université de Sherbrooke, CANADA

## Abstract

Atmospheric N deposition is predicted to increase four times over its current status in tropical forests by 2030. Our ability to understand the effects of N enrichment on C and N cycles is being challenged by the large heterogeneity of the tropical forest biome. The specific response will depend on the forest’s nutrient status; however, few studies of N addition appear to incorporate the nutrient status in tropical forests, possibly due to difficulties in explaining how this status is maintained. We used a meta-analysis to explore the consequences of the N enrichment on C and N cycles in tropical montane and lowland forests. We tracked changes in aboveground and belowground plant C and N and in mineral soil in response to N addition. We found an increasing trend of plant biomass in montane forests, but not in lowland forests, as well as a greater increase in NO emission in montane forest compared with lowland forest. The N_2_O and NO emission increase in both forest; however, the N_2_O increase in lowland forest was significantly even at first time N addition. The NO emission increase showed be greater at first term compared with long term N addition. Moreover, the increase in total soil N, ammonium, microbial N, and dissolved N concentration under N enrichment indicates a rich N status of lowland forests. The available evidence of N addition experiments shows that the lowland forest is richer in N than montane forests. Finally, the greater increase in N leaching and N gas emission highlights the importance of study the N deposition effect on the global climate change.

## Introduction

Atmospheric nitrogen (N) deposition has increased globally by a factor of 3.6 since the pre-industrial era due to fertilizer applications, fossil fuel combustion, and legume cultivation [1]. Despite the fact that there is substantial uncertainty about N production [2], the increase in anthropogenic derivate N in the tropical region is similar to or exceeds the rates of biological fixation before human activity became the largest source of new N in the biosphere [3, 4]. Our ability to predict responses to anthropogenic N addition is challenged by the complexity of forest biomes. Evidence from a wide variety of terrestrial ecosystems suggests that increases in N affect carbon (C) and N cycles in a coupled way, since organisms require C, N, and P in specific proportions, from the molecular to the global scale [5, 6, 7, 8]. The incorporation of this ecological stoichiometric approach is relevant for understanding future changes in both global nutrient cycles and the climate [8, 9, 10]. Recent climate models with C cycle component, that incorporate N deposition as a part of the CN-coupled models predict a lower net C uptake (37–74%) than values projected by models using C cycle components alone, demonstrating the importance of the N cycle in future climate change [9, 10].

Tropical forests (TFs) are among the most important biomes on Earth, globally supporting one-third of the annual terrestrial net primary productivity and storing about 25% of the world’s biomass C [11, 12]. Together, these characteristics and the complexity of the tropical biomes make it difficult to achieve a complete understanding about the relationships between anthropogenic N addition and C cycling and their impact on global change [8, 9, 10, 11, 13]. Despite their importance, fundamental uncertainties remain in our understanding of the effect of the worldwide anthropogenic N addition on the C and nutrient cycles of these forests [14].

The best test to understand the link between the C and N cycles in the context of N enrichment is through the direct manipulation of the N supply [14]. However, in TFs, it is difficult to interpret the diverse results because they are typically derived from a few heterogeneous locations with different experimental designs. Consequently, there is still high uncertainty regarding patterns and mechanisms that explain the interactions among C, N, and P elements with an increase in N deposition [15, 16]. TF biome ecosystems generally grow on old and highly weathered soils in which the soil N tends to accumulate by biological N fixation [14, 17]. Nonetheless, TFs are classified as either N-limited montane forests or richer lowland forests [12, 14]. This classification scheme has motivated a trend in research toward the expectation that lowland forests do not exhibit a biological growth response to N increase, and lowland forests are even recognized as N-saturated ecosystems [12, 17]. The concept of N saturation refers to the idea that when N supply exceeds biological demand, then new N is lost from the ecosystem by biological or physical means [17]. It is difficult to reconcile these and similar broad trends with a clear pattern of TFs’ N status and the effect of anthropogenic N addition. The lack of sufficient data from N addition experiments in TFs and the difficulty in discriminating among different groups of ecosystems in TF biomes have resulted in a remarkable underrepresentation when compared to non-tropical forests in other synthetic analyses (i.e., they comprise ~5 to 12% of studies) [5, 16, 18]. Usually, experimental N addition in TFs are performed under conditions that do not fit the criteria used in synthetic analyses; for instance, the duration of N addition is typically for a period of less than one year. In addition, the plot sizes for the experimental manipulation can be so small that they do not exclude edge effects. On the other hand, some studies use pot experiments that entail ecosystem disturbance and separation from actual field conditions that can introduce artificial effects. For this paper, resolving the uncertainty in TFs required accepting a few assumptions.

We assumed that TFs occur along a continuum of N availability from N limited in young and lightly weathered soils in montane elevation forests to N non-limited in old substrate landscapes, lowland elevations, and mature forests [19, 20, 21]. We also recognized the evidence that suggests that even in mature lowland TFs, there are micro-sites in which biological activity is N limited or where co-limitations between N, P, and K have been found [22–23]. Here, we report the results of a meta-analysis carried out to clarify the N addition effect on C and N cycles in TFs. We focus on TFs identified as lowland (< 1,000 m asl) and montane (> 1,000 m asl) forests to understand whether the N status influences the response of C, N, and P to N addition.

Our purpose here is to synthesize the research related to the consequences of N addition. Specifically, we compared C and N cycle changes in montane and lowland TFs using a currently available group of aboveground plant variables and belowground variables as organic and mineral soil variables. We expect that montane and lowland tropical forests respond to anthropogenic N with changes in the C and N cycles, with an increase in plant biomass without changes in N loss in montane forests and increase in N loss in lowland forest. In contrast, in lowland forests, this increase might weakly affect forest biomass and increase N loss. We also analyze how the rate and duration of N addition and the chemical form of the N added influence the C and N cycles’ response.

## Methods

### Data Compilation and Classification

We searched the literature to identify quantitative studies on the effects of N addition on soil C and N cycles. We used the ISI Web of Science database (http://apps.isiknowledge.com), as it provides access to peer-reviewed literature. The search comprised studies published between 1985 and 2014 that included *tropical forest* and *subtropical forest* as keywords. Preliminary results were filtered to include only research focused on nitrogen, nitrogen addition, nitrogen fertilization, and nitrogen deposition. An ISI-defined area was used to filter our search. We only dealt with studies that explicitly considered (1) the chemical form of N added; (2) the N addition rate and the duration of the experimental period, and (3) the means and sample sizes associated with the selected variables for the control and N-supply treatments. Overall, in the final data compilation, the number of studies initially considered was restricted based on different criteria. First, only studies that reported at least one C or N pool or flux were used. Second, subtropical forest studies were refiltered focusing on forests without freezing conditions. Third, only studies with more than 5 x 5 m plots were included. Finally, only studies with field-based measurement were included. We constructed a database for 35 response variables (S1 Table) in montane and in lowland TFs; statistical information was recorded to compare each pair control and treatment (“*k* pair”). For instance, when a study considered three treatments (t1, t2, and control), the data were reorganized in two pairs: t1 versus control and t2 versus control. The statistical information included sample size, mean, and standard error or deviation. Unlabeled error bars were assumed to denote the standard error and were changed to standard deviation. We used DigitizeIt 1.5 (GeoMem Software, Perthshire, UK) to extract data from figures.

To detect the effects of experimental conditions on response variables, the rate and duration of the N supply and chemical form of the N added were considered as modulator variables. The experimental condition variables included the N addition rate (kg N ha/year^-1^; low, < 75; medium, 75–125; high, >125), time of N supply (in months; first term (<13), intermediate term (13–35); long term (>35), and chemical form of the N added (urea; urea+NH_4_NO_3_; NH_4_NO_3_; others). We considered the site features, including the ecosystem, elevation, and vegetation successional stage.

In studies with multiple time points or soil horizons, only data on final time and/or “A horizon” were considered in order to avoid interdependence between analyzed values for the same variable. However, measurements within a study using different levels of N supply, chemical for of the N added, and sites with different environmental conditions were considered independent.

### Meta-Data Analysis

The meta-analysis was performed following Hedges et al.’s procedure [24], using MetaWin 2.0 software [25]. For each combination of response variable and type of TF, we used the natural log of response ratio (lnR) to calculate the effect size, which is a proportion of the change resulting from experimental manipulation. The first step in the analysis was to calculate the weighted lnR and its variance for all paired observations for each response variable. Then, these values were used to fit four models to estimate the N-addition effect through the main lnR and its confidence intervals (CIs). A principal model was used to perform a general analysis exploring the responses of lowland and mountain TFs and to identify significant differences between them, and three complementary analyses included each experimental variable and identified significant differences in these variables between the forest types.

The models incorporated a mixed approach when at least two categories in each modulator variable (i.e., type of TF or each experimental variable) had at least three *k* pairs; otherwise, the models used a fixed approach. There are two important statistics in these models: the *p*-value associated with a fixed component (i.e., total heterogeneity (Qt)), which is significant when all data variance was explained by the model, and the *p*-value associated with a random component (*p-*value Ran; i.e., between-group heterogeneity (Qb)), which is significant when different responses exist among modulator variable categories [26]. A non-significant Qt was interpreted as representing a need to incorporate more modulator variables to explain all the heterogeneity. CIs calculated from each model were used to determine the statistical effect of N addition on each variable; the N-addition effect was considered significant when a 95% CI did not include zero. The percentage change of significant variables was calculated from (R–1)*100, where a value other than 0 indicated an increase or decrease of the response variable in response to the N supply.

## Results

**Fig 1:**
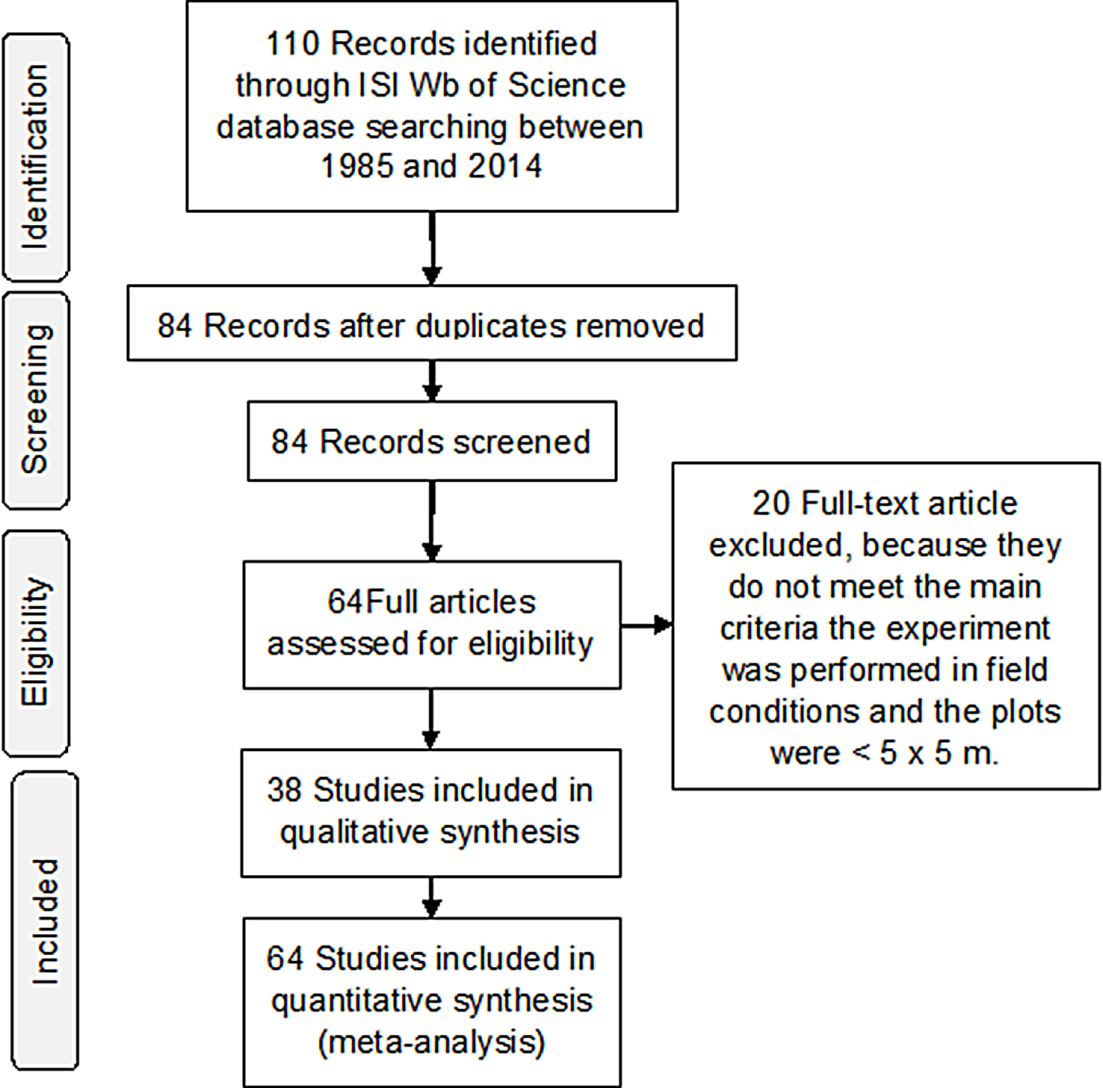
Summary of database searching and inclusion of final groups.

**Fig 2:**
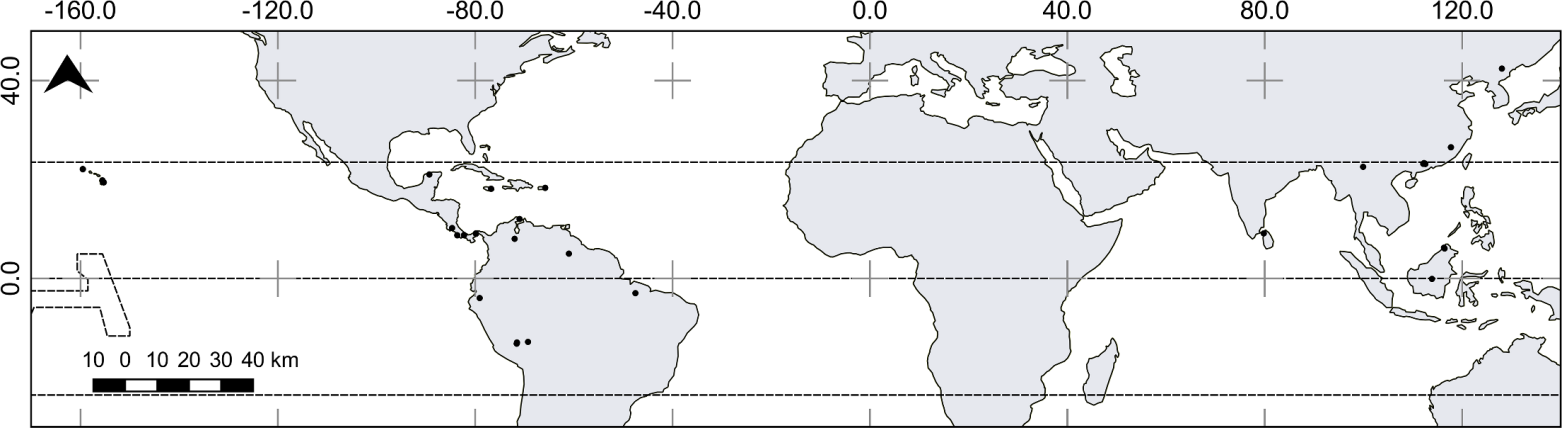
Localities included in the meta-analysis. Made with Natural Earth. Free vector and raster map data @ naturalearthdata.com.

In total, 84 references documenting the effects of N addition on C and N cycles in tropical forests were included (Fig 1). Abstracts and titles of these publications were reviewed, and 21 were discarded because they did not meet the eligibility criteria. Therefore, 64 studies that contained appropriate subject matter remained (S1 Text). The final dataset comprised 64 publications, and the studies were distributed across 39 locations in 14 countries (Fig 2); ~30% of the total research studies were performed in Hawaii, and the other 25% were performed in China. The representations of lowland and montane tropical forests were equal in the studies analyzed (i.e., 20 locations in lowland and 21 locations in montane tropical forest). The total number of *k* comparisons used in the analysis was 759. In experiments, the most common conditions were medium-rate, long-term NH_4_NO_3_ addition (S1 Fig). The lack of P data impeded making a conclusion on this nutrient cycle.

### Aboveground C and N Pools and Fluxes

**Fig 3:**
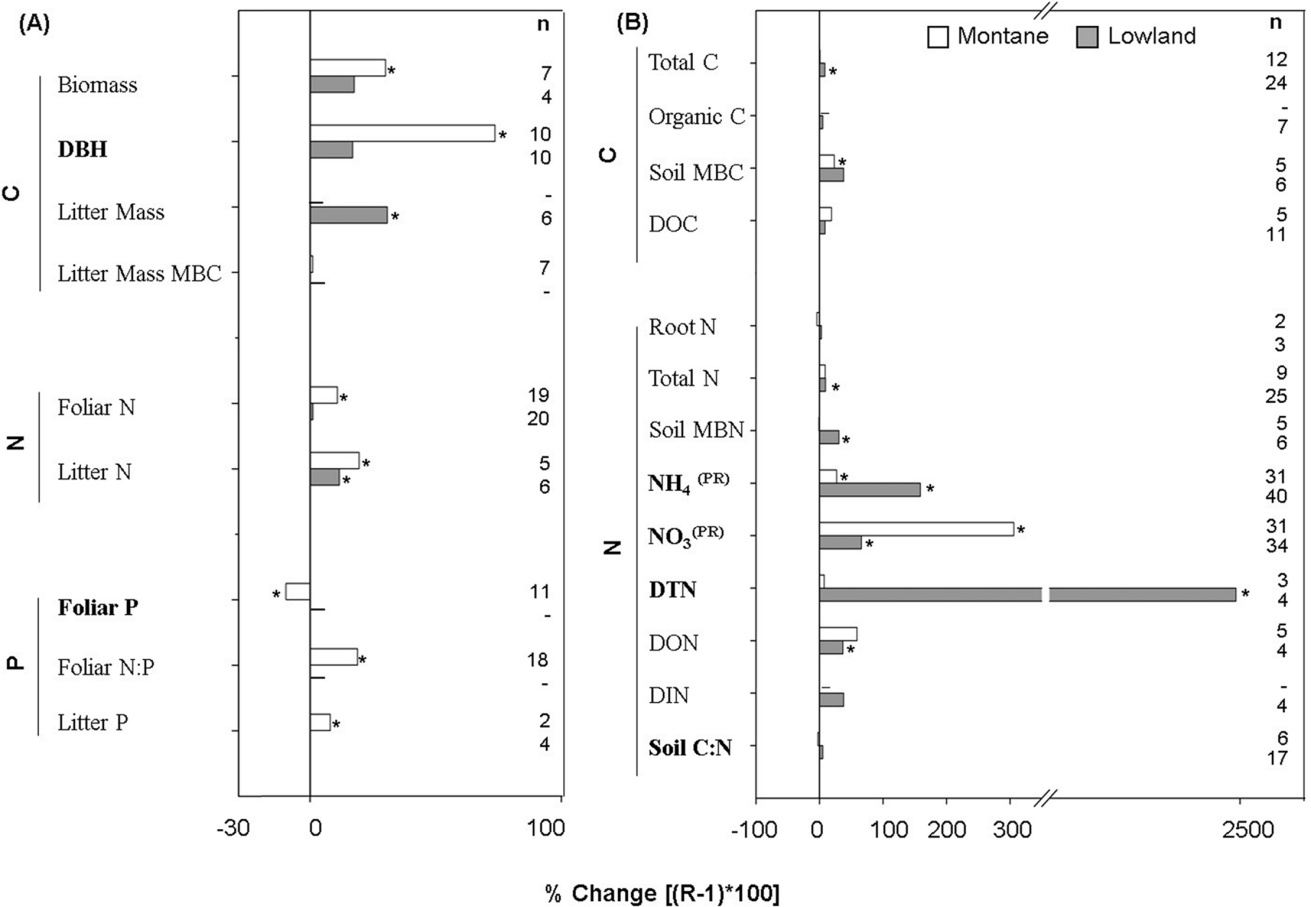
Percentage of change for response pools in (A) aboveground and (B) belowground variables. An asterisk represents significant percentage of change with the N addition. A PR superscript represent a significantly different change between montane and lowland forests (ProRand ≤ 0.05). Bolded variables represent significant associated total heterogeneity (Qt; p ≤ 0.05). n represent the number of pair comparison between control and experimental N-supply.

**Fig 4:**
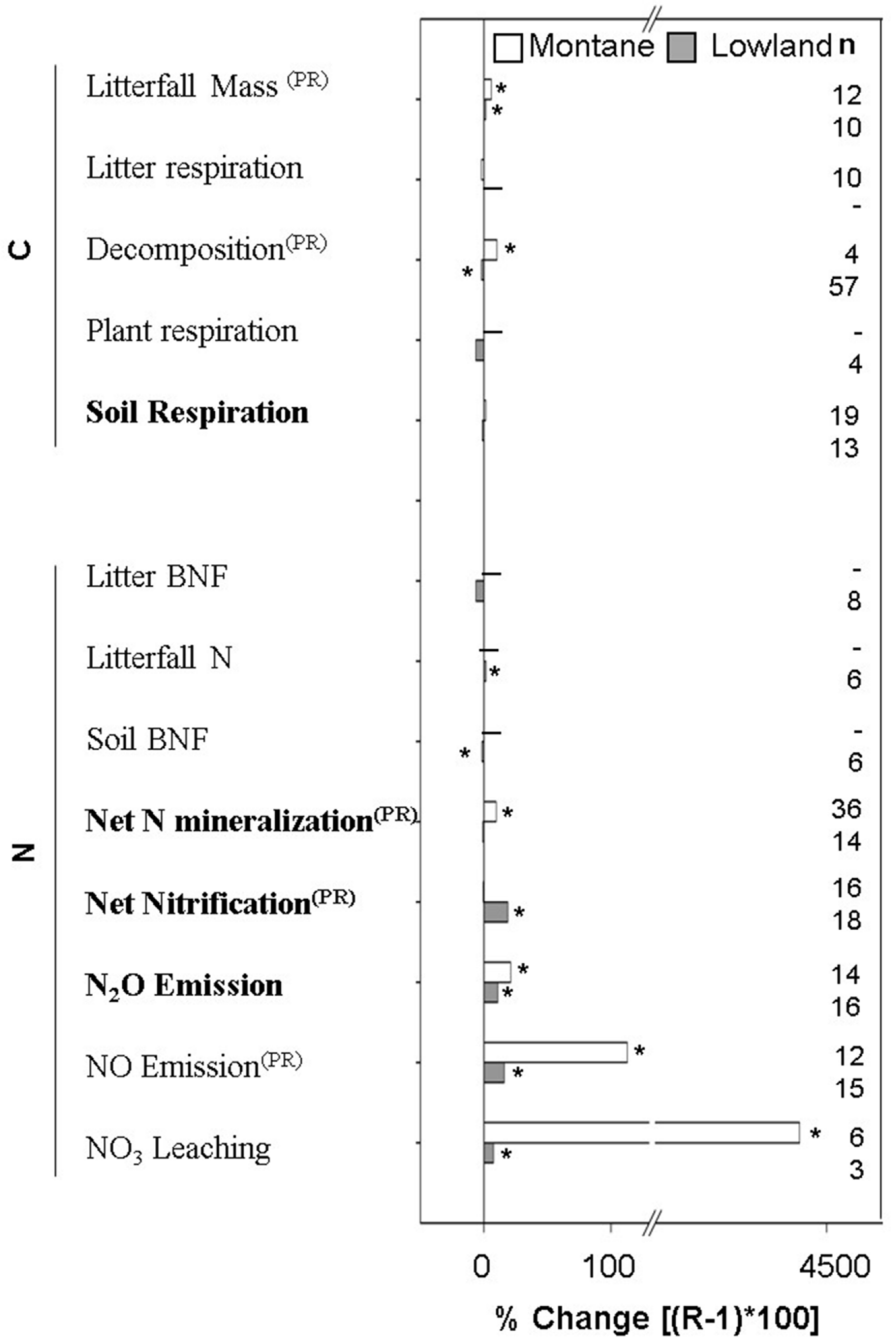
Percentage of change for flux variables. An asterisk (*) represents a significant percentage of change with the N addition. A PR superscript indicates significantly different change between montane and lowland forests (ProRand ≤ 0.05). Bolded variables represent significant associated total heterogeneity (Qt; p ≤ 0.05).

Our results showed a general trend of an increase in pools and fluxes of C and N cycles in montane and lowland forests. Only a few variables decreased significantly with N addition, that is, soil biological N fixation (BNF) and decomposition in lowland forests (Fig 3A and Fig 4). The biomass and diameter at breast height (DBH) increased in montane forests (Fig 3A), and the litterfall mass increased consistently in both lowland and montane forests, with a major increase in montane forests (ProRand < 0.05; Fig 4). The increase of foliar N was significant in montane forests exclusively. In contrast, litter N increased significantly in lowland and montane forests (Fig 3A). Similarly, litter P increased in montane forests. The lack of foliar P and foliar N:P data for lowland forests did not allow a comparison between forest types (Fig 3A).

### Belowground C and N Pools and Fluxes

Nitrogen addition affected almost 60% of the examined belowground pools variables (Fig 3B). In lowland forests, soil total C, soil total dissolved N (TDN), and soil dissolved organic N (DON) increased, and no change was seen in montane forest (Fig 3B). In contrast, soil microbial biomass carbon (MBC) increased significantly in montane forests, and no change was seen in lowland forests. The increase in dissolved organic C (DOC) in montane and lowland forests was not significant (Fig 3B). We did not find effects of N addition on root N, dissolved inorganic N (DIN), and the soil C:N ratio (Fig 3B). The total N, soil microbial N mass (MBN), and dissolved organic N increased in lowland forests exclusively (Fig 3B). The increase in soil inorganic N was statistically different in montane and lowland forests (ProRand = 0.03 and 0.04, respectively; Fig 3B). The increase in soil nitrate (NO_3_^-^) was higher in montane forests. Conversely, the increase in ammonium (NH_4_^+^) was higher in lowland forests. The lack of litter BNF, litter N, and soil BNF data for montane forests impeded the comparison between TFs.

Decomposition increased in montane forests and decreased in lowland forests (ProRand = 0.012; Fig 4). Changes in soil respiration were not significant in montane and lowland forest (Fig 4). Nitrogen fluxes escalated because of increasing net N mineralization in montane forests, and there was no change in lowland forests (ProRand = 0.001; Fig 4). In contrast, net nitrification increased significantly in lowland forests, but there was no change in montane forests (ProRand = 0.007; Fig 4). Whit N addition, N_2_O and NO emissions increased in montane and lowland forests. The increase in NO emissions was significantly higher in montane than in lowland forests (ProRand = 0.001; Fig 4). Finally, NO_3_^-^ leaching increased dramatically in both montane and lowland forests.

### Effects of the Experimental Conditions

**Fig 5:**
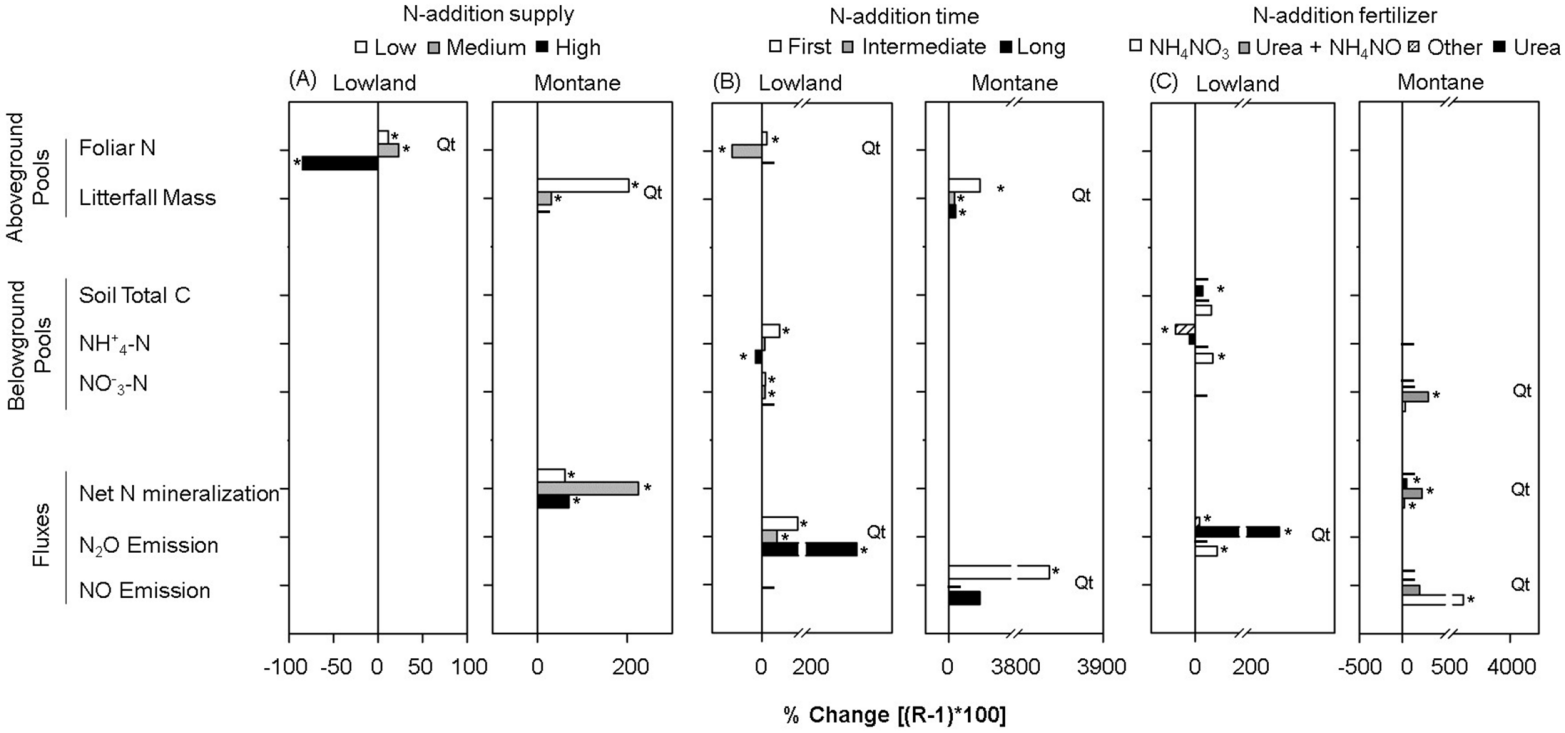
Percentage of change for variables responding in different ways to the different experimental conditions. An asterisk represents a significant percentage of change with the N addition. *Qt* is the significance (p ≤ 0.05) of associated total heterogeneity (Qt). (A) N- addition supply, (B) N-addition time and (C) N-addition fertilizer.

Approximately 40% of the examined studies were carried out with a high N addition rate (S1A Fig). In lowland forests, the foliar N decreased significantly with high rates of N addition (i.e., > 125 kg N ha yr^-1^), which contrasted with the increase observed at medium and low rates of N addition (ProRand = 0.002; Fig 5). Other response variables did not respond to the three levels of N addition. In montane forests, litterfall and net N mineralization increased with low (i.e., < 75–125 kg N ha yr^-1^) and medium (i.e., 75–125 125 kg N ha yr-1) rates of N addition (Fig 5), respectively. In addition, net N mineralization increased with a high (i.e., > 125 kg N ha yr^-1^) rate of N addition (Fig 5). The litterfall mass increase was greater in experiments with low N-addition rates (ProRand = 0.01; Fig 5), and there was no data to compare the results under high N-addition rates.

There were insufficient data to examine the impact of forest successional stage and forest composition on C and N cycles in montane and lowland forests.

Approximately 30% of the studies examined were carried out with long-term (i.e., >36 month) N-addition exposure (S1B Fig). In lowland forests, foliar N decreased significantly with the intermediate duration (13–35 months) of N-addition exposure (Fig 5); in contrast, it increased with the first duration (<13 months) of N-addition exposure (ProRand = 0.001; Fig 5). Inorganic N increased with first and intermediate durations of N-addition exposure (Fig 5); however, ammonium (NH_4_^+^-N) decreased with long-term N-addition exposure (>35 months), and there were no data on NO_3_^—^N with high N-addition exposure. The N_2_O emissions were greater with long-term N-addition exposure (>35 months) compared with intermediate- and first-term N addition (ProRand = 0.004) (Fig 5). In montane forests, litterfall-mass production increased with N-addition exposure time; this increase was greater with first-term than with long- and intermediate-term N additions (ProRand = 0.01). Similarly, NO emissions increased significantly with long-term N addition (ProRand = 0.011), and no significant response was found with first- and intermediate-term N additions (Fig 5).

The N addition in the majority of the examined studies used NH_4_^+^NO_3_^-^ (~40%) (S1C Fig). The fertilizer effect was significant on belowground variables alone. In lowland forests, soil total C increased with urea N addition (Fig 5). There was no significant response to other fertilizer types. The soil NH_4_ increased when NH_4_NO_3_ was applied (ProRand = 0.002); with another type of fertilizer, NH_4_ decreased (Fig 5). The N_2_O increase was four times higher with urea compared with NH_4_^+^NO_3_^-^ and other fertilizer types, and there were no data for urea + NH_4_^+^NO_3_^-^. In montane forests, soil NO_3_-N, net N mineralization, and NO emissions increased at different magnitudes with differing fertilizer types (ProRand = 0.008; 0.002, and 0.016, respectively). The highest increase was found in NO emissions when NH_4_^+^NO_3_^-^ was used (Fig 5).

## Discussion

### Effects of N Addition: Montane versus Lowland Forests

The available data offer the opportunity to track the C and N response to N addition in montane and lowland forests and are useful in synthesizing the relevant information about the bioavailability of C and N in tropical forests. The available evidence suggested that montane forests is N limited, because plants growth (i.e., where biomass and DBH increases, as shown in Fig 2), the productivity increased (litterfall mass), and the foliar N increased; however, their results were in contrast with the increase in the losses of bioavailable N (NO_3_^-^ leaching and NO and N_2_O emission). On the other hand, lowland forests did not show evidence of plant growth, and similarly to montane forests, they had high losses of bioavailable N and gas emissions. Because the N loss has been considered as a direct signal of forest non-N limitation [14, 27], the evidence here analyzed supports the non-N limitation in montane and lowland tropical forests. These results suggest that anthropogenic N deposition delivers sufficient external N to tropical biomes in concordance with the atmospheric nitrogen deposition hypothesis of Hedin et al. [14]. In agreement with the generally accepted idea, our additional observations of soil N levels in lowland forests (i.e., the increase of total soil N, soil microbial biomass N, dissolved total N, and ammonium) indicate the initially rich N status in lowland forests. The mechanisms of soil N retention in lowland forests are not resolved with the present analysis. In the same way, the increase in decomposition in montane forests, in an opposite manner to the decrease in lowland forests, and the net N mineralization increase in the montane forests without any change in lowland forests provide support for the notion that lowland forests are comparably richer in N than montane forests. Additional observations showed that N enrichment in montane forests might increase the soil microorganism’s biomass, in contrast with previous reviews using results of temperate forest, where the microbial biomass decrease with the N addition [28]. The response of microbial biomass to the N addition is poorly represented in tropical forest (here we included six studies), thus their apparent N limitation in montane tropical forest required additional research. Interestingly, decomposition and litterfall-mass production changed in both montane and lowland forests in response to N addition. These C fluxes played a central role in ecosystem functioning [29] and help to explain the more realistic results for climate projections obtained using component of C and N than with only C cycle [9, 10]. The decline in decomposition in lowland forests by N addition is in concordance with the results reported by Knorr et al. [30]. Despite the fact that the mechanism of such pattern in lowland forests has been explained based on microbial community composition and their production of extracellular enzymes [28, 31, 32], as well as by the synthesis of new toxic N compounds [33], it is urgent to clarify mechanisms that explain different pathways of decomposition changes in montane and lowland forests. The decomposition increase in montane forest could affect forest productivity and limit the potential to mitigate climate change by accelerate global warming [34].

Our results suggest that the major increase of ammonium concentration in lowland forests can be favoring the increases in net nitrification, but the cost of this pathway appears to be the loss of NO_3_^-^ through leaching. In the contrasting case, the low increase of ammonium in montane forests and high net N mineralization without any change in net nitrification suggest that ammonium is immobilized into biological tissues, principally in foliar tissues, as is evidenced by the increase of foliar N [29]. By the other way, in montane forest the inorganic N remaining in the soil (i.e., NO_3_^-^) can to favor the N loss. The increase in NO and N_2_O emissions, supports the last proposition, when it is exposed to high-rate and long-term N addition, as seen in other TFs [17]. The N added could be used by bacteria, which mediates the N loss [35, 36].

### Trends in the Effects of N Enrichment due to Experimental Conditions

Our results demonstrated that in lowland forests, N addition had an almost significantly positive effect on foliar N when the rate of N addition did not exceed 125 Kg N ha^-1^ yr^-1^ (i.e., high) and when the duration of N addition was not greater than 13 months (i.e., first term). When such limits are exceeded, foliar N decreases, which indicates that plants cannot absorb the excess N in conditions with chronic N addition. The response of N_2_O loss to N-addition exposure time was non-accumulative and lineal across the first (<13 months), intermediate (13–35 months), and long-term (>35 month) durations. In this context, regardless of nutrient status, lowland tropical forests responded during the first year of N addition; N_2_O gas emission initially decreased and then finally increased substantially over the 35 months of experiments. The lack of change in aboveground variables with different chemical of the N for added is in accord with previously obtained results to a global scale [18].

Additional observations of the increase in litter and litterfall N might appear to be decoupled from foliar N decreases under experimental conditions simulating chronic N deposition. However, this last effect was observed under longer-term N experimental conditions (~33%) than the conditions present for the first term (12%), demonstrating that our analyses have uncertainties associated with the unequal representation of the experimental condition categories.

### Conclusions

Our analysis indicates that an experimental N deposition exerts significant control over the mechanisms that mediate N loss across montane and lowland tropical forests. Our findings also indicate that the available evidence from studies on the effect of N deposition, which use several individual variables, makes it difficult to interpret the N status in montane and lowland tropical forests. The last is in part because the evidence is derived from a few locations but also because of the lack of concordance in terms of the key variables to be measured. Given that human activity has doubled the amount of reactive N circulating in the terrestrial biosphere [37], our understanding of the linkages between the N input and C and N cycles and their relationship with the forest function [30] should challenge researchers to design more realistic climate models by use components of C and N cycles [9, 10]. Because the increase in N gas emissions is a common change in montane and lowland forests, we can expect that N deposition could influence climate by increasing global warming [38]. In the same way, the increase of N leaching in montane and lowland forests can have an impact in the eutrophication of aquatic ecosystems in TFs [15, 38].

## Supporting Information

S1 Fig Frequency distributions according to experimental conditions used in this meta-analysis. (DOCX)

S1 Table 35 response variables considered in this study (DOCX)

S1 Text List of 64 papers from which data were extracted for this meta-analysis (DOC)
