# Supplementary material for: Effects of Increased Nitrogen Availability on C and N Cycles in Tropical Forests: A Meta-Analysis
Source: PLoS One. 2015 Dec 3;10(12):e0144253. doi: 10.1371/journal.pone.0144253 (PMC4669154; doi:10.1371/journal.pone.0144253)
Supplement: S1 Fig — (DOCX) [file pone.0144253.s001.docx]

**S1 Fig. Frequency distributions according to experimental conditions used in this meta-analysis**

**
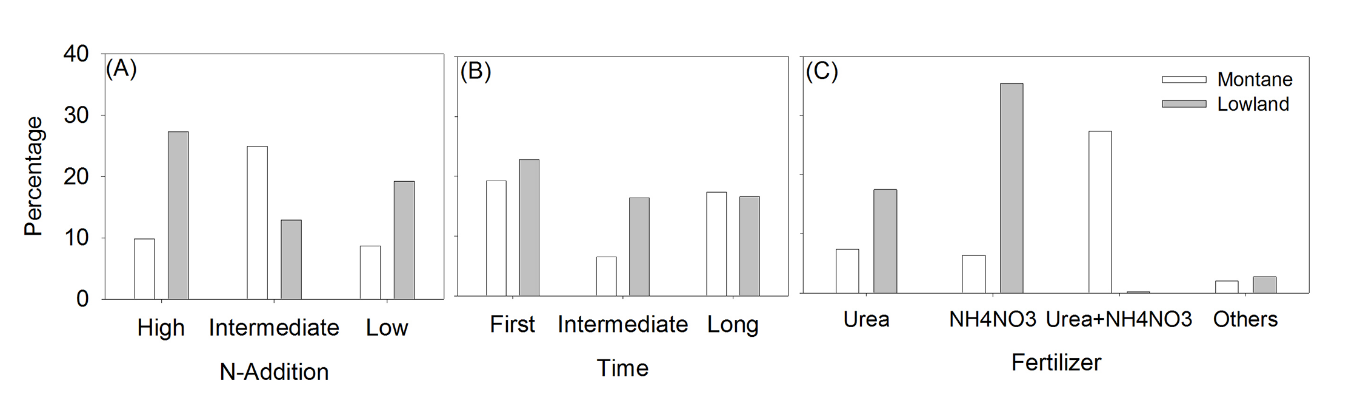
**
