# Supplementary material for: Effects of Increased Nitrogen Availability on C and N Cycles in Tropical Forests: A Meta-Analysis
Source: PLoS One. 2015 Dec 3;10(12):e0144253. doi: 10.1371/journal.pone.0144253 (PMC4669154; doi:10.1371/journal.pone.0144253)
Supplement: S1 Table — (DOCX) [file pone.0144253.s002.docx]

**S1. Table 1.** 35 response variables considered in this study

| **Pools/Fluxes** | **Bio-element** | **Response variable** | **Observations** |
| --- | --- | --- | --- |
| **Aboveground** | | | |
| **Pools** | **C** | Biomass | Montane/Lowland |
|  |  | Diameter at breast height (DBH) | Montane/Lowland |
|  |  | Litter mass | Lowland |
|  |  | Litter mass microbial biomass carbon (MBC) | Montane |
|  | **N** | Foliar N | Montane/Lowland |
|  |  | Litter N | Montane/Lowland |
|  | **P** | Foliar P | Montane |
|  |  | Litter P | Montane/Lowland |
|  |  | Foliar N:P | Montane |
| **Fluxes** | **C** | Plant Respiration | Lowland |
|  |  | Litterfall Mass | Montane/Lowland |
|  |  | Litter Respiration | Montane |
|  | **N** | Litter biological nitrogen fixation (BNF) | Lowland |
|  |  | Litterfall N | Lowland |
| **Belowground** | | | |
| **Pools** | **C** | Total C | Montane/Lowland |
|  |  | Organic C | Lowland |
|  |  | Soil microbial biomass carbon (SMBC) | Montane/Lowland |
|  |  | Dissolved organic carbon (DOC) | Montane/Lowland |
|  | **N** | Root N | Montane/Lowland |
|  |  | Total N | Montane/Lowland |
|  |  | Soil microbial biomass nitrogen (SMBN) | Montane/Lowland |
|  |  | Ammonium (NH_4_) | Montane/Lowland |
|  |  | Nitrate (NO_3_) | Montane/Lowland |
|  |  | Dissolve total nitrogen (DTN) | Montane/Lowland |
|  |  | Dissolved organic nitrogen (DON) | Montane/Lowland |
|  |  | Dissolved inorganic nitrogen (DIN) | Lowland |
|  |  | Soil C:N | Montane/Lowland |
| **Fluxes** | **C** | Decomposition | Montane/Lowland |
|  |  | Soil Respiration | Montane/Lowland |
|  | **N** | Soil biological nitrogen fixation (BNF) | Lowland |
|  |  | Net Mineralization | Montane/Lowland |
|  |  | Net Nitrification | Montane/Lowland |
|  |  | N_2_O Emission | Montane/Lowland |
|  |  | NO Emission | Montane/Lowland |
|  |  | NO_3_ leaching | Montane/Lowland |
