# Supplementary material for: Effects of Increased Nitrogen Availability on C and N Cycles in Tropical Forests: A Meta-Analysis
Source: PLoS One. 2015 Dec 3;10(12):e0144253. doi: 10.1371/journal.pone.0144253 (PMC4669154; doi:10.1371/journal.pone.0144253)
Supplement: S1 Text — (DOC) [file pone.0144253.s003.doc]

**Supporting information**

**S1.** L**ist of 64 papers from which data were extracted for this meta-analysis**

1. Adamek M, Corre MD, Holscher D (2009) Early effect of elevated nitrogen input on above-ground net primary production of a lower montane rain forest, Panama. Journal of Tropical Ecology 25: 637–647
2. Alvarez-Clare S, Mack Mc, Brooks M (2013) A direct test of nitrogen and phosphorus limitation to net primary productivity in a lowland tropical wet forest. Ecology, 94(7): 1540–1551
3. Andersen KM, Corre MD, Turner BL, Dalling JW (2010) Plant–soil associations in a lower montane tropical forest: physiological acclimation and herbivore‐mediated responses to nitrogen addition. Functional Ecology 24: 1171–1180
4. Balser TC (2001) The impact of long-term nitrogen addition on microbial community composition in three Hawaiian forest soils. The Scientific World 1: 500–504
5. Bejarano M, Etchevers JD, Ruíz-Suárez G, Campo J (2014) The effects of increased N input on soil C and N dynamics in seasonally dry tropical forests: an experimental approach. Applied Soil Ecology 73: 105-115
6. Cai ZQ, Poorter L, Han Q, Bongers F (2008) Effects of light and nutrients on seedlings of tropical Bauhinia lianas and trees. Tree Physiology 28: 1277–1285
7. Campo J, Dirzo R (2003) Leaf quality and herbivory responses to soil nutrient addition in secondary tropical dry forests of Yucatan Mexico. Journal of Tropical Ecology 19: 525–530
8. Campo J, Solís E, Valencia MG (2007) Litter N and P dynamics in two secondary tropical dry forests after relaxation of nutrient availability constraints. Forest Ecology and Management 252: 22–40
9. Campo J, Vázquez-Yanes C (2004) Effects of nutrient limitation on aboveground carbon dynamics during tropical dry forest regeneration in Yucatán Mexico. Ecosystems 7: 311–319
10. Cavelier J, Tanner E, Santamaria J (2000) Effect of water temperature and fertilizers on soil nitrogen net transformations and tree growth in an elfin cloud forest of Colombia. Journal of Tropical Ecology 16: 83–99
11. Chen H, Dong S, Liu L, Ma C, Zhang T, Zhu X, Mo J (2013) Effects of Experimental Nitrogen and Phosphorus Addition on Litter Decomposition in an Old-Growth Tropical Forest. PlosOne e84101 doi:10.1371/journal.pone.0084101
12. Cleveland CC, Reed SC, Townsend AR (2006) Nutrient regulation of organic matter decomposition in a tropical rain forest. Ecology 87: 492–503
13. Cleveland CC, Townsend A (2006) Nutrient additions to a tropical rain forest drive substantial soil carbon dioxide losses to the atmosphere. PNAS 103 10316–10321
14. Corre MD, Veldkamp E, Arnold J, Wright SJ (2010) Impact of elevated N input on soil N cycling and losses in old-growth lowland and montane forests in Panama. Ecology 91: 1715–1729
15. Cusack DF, Torn MS, McDowell WH, Silver WL (2010) The response of heterotrophic activity and carbon cycling to nitrogen additions and warming in two tropical soils. Global Change Biology 16 2555–2572
16. Cusack D, Silver WL, Torn MS, McDowell WH (2011) Effects of nitrogen additions on above- and belowground carbon dynamics in two tropical forests. Biogeochemistry 104: 203–225
17. Davidson EA, Reis de Carvalho CJ, Vieira ICG, Figueiredo RO, Moutinho P, Ishida Y, et al (2004) Nitrogen and phosphorus limitation of biomass growth in a tropical secondary forest. Ecological Applications 14: 150–163
18. Fan H, Wu J, Liu W, Yuan Y, Huang R, Liao Y, Li Y. (2014) Nitrogen deposition promotes ecosystem carbon accumulation by reducing soil carbon emission in a subtropical forest Plant Soil 379:361–371
19. Fang HJ, Mo J, Peng S, Li Z, Wang H (2007) Cumulative effects of nitrogen additions on litter decomposition in three tropical forests in southern China. Plant and Soil 297: 233–242
20. Fang Y, Zhu W, Gundersen P, Mo J, Zhou G, Yoh M (2009) Large loss of dissolved organic nitrogen from nitrogen-saturated forests in subtropical China. Ecosystems 12: 33–45
21. Fang Y, Zhu W, Mo J, Zhou G, Gundersen P (2006) Dynamics of soil inorganic nitrogen and their responses to nitrogen additions in three subtropical forests south China. Journal of Environmental Sciences-China 18: 752–759
22. Fisher JB, van de Weg MJ, Malhi Y, Meir P, Torres IC, Silva-Espejo JE, Metcalfe DB, Huasco WH (2012) Nutrient limitation in rainforests and cloud forests along a 3,000-m elevation gradient in the Peruvian Andes. Oecologia DOI 10.1007/s00442-012-2522-6
23. Gamboa AM, Hidalgo C, De León F, Etchevers JD, Gallargo JF, Campo J (2010) Nutrient addition differentially affects soil carbon sequestration in secondary tropical dry forests: Early-versus late succession stages. Restoration Ecology 18: 252–260
24. Gower ST, Vitousek P (1989) Effects of nutrient amendments on fine root biomass in a primary successional forest in Hawai'i. Oecologia 81 566-568
25. Hall SJ, Asner GP, Kitayama K (2004) Substrate climate and land use controls over soil N dynamics and N-oxide emissions in Borneo. Biogeochemistry 70: 27–58
26. Hall SJ, Matson PA (1999) Nitrogen oxide emissions after nitrogen additions in tropical forests. Nature 400: 152–155
27. Hall SJ, Matson PA (2003) Nutrient status of tropical rain forests influences soil N dynamics after N additions. Ecological Monographs 73: 107–129
28. Harrington RA, Fownes JH, Vitousek PM (2001) Production and resource use efficiencies in N-and P-limited tropical forests: A comparison of responses to long-term fertilization. Ecosystems 4: 646–657
29. Herbert DA, Fownes JH (1995) Phosphorus limitation of forest leaf area and net primary production on a highly weathered soil. Biogeochemistry 29: 223–235
30. Herbert DA, Fownes JH (1999) Forest productivity and efficiency of resource use across a chronosequence of tropical montane soils. Ecosystems 2: 242–254
31. Kaspari M, Milton N, Harms KE, Santana M, Wright SJ, Yavitt JB (2008) Multiple nutrients limit litterfall and decomposition in a tropical forest. Ecology Letters 11: 35–43
32. Koehler B, Corre MD, Veldkamp E, Sueta JP (2009) Chronic nitrogen addition causes a reduction in soil carbon dioxide efflux during the high stem-growth period in a tropical montane forest but no response from a tropical lowland forest on a decadal time scale. Biogeosciences 6: 2973–2983
33. Krashevska V, Maraun M, Ruess L, Scheu S (2010) Carbon and nutrient limitation of soil microorganisms and microbial grazers in a tropical montane rain forest. Oikos 119: 1020–1028
34. Li X, Zheng X, Han S, Zheng J. Li T (2010) Effects of nitrogen additions on nitrogen resorption and use efficiencies and foliar litterfall of six tree species in a mixed birch and poplar forest, northeastern China. Canadian Journal of Forest Research 40 2256–2261
35. Liu J, Xu Z, Zhang D, Zhou G, Deng Q, Duan H, et al. (2011) Effects of carbon dioxide enrichment and nitrogen addition on inorganic carbon leaching in subtropical model forest ecosystems. Ecosystems 14: 683–697
36. Liu L, Zhang T, Gilliam FS, Gundersen P, Zhang W, Chen H, Mo J (2013) Interactive Effects of Nitrogen and Phosphorus on Soil Microbial Communities in a Tropical Forest. PLoS ONE 8: e61188. doi:10.1371/journal.pone.0061188
37. Lohse KA, Matson P (2005) Consequences of nitrogen additions for soil losses from wet tropical forests. Ecological Applications 15: 1629–1648
38. Lu X, Mo J, Gilliam FS, Fang H, Zhu F, Fang Y, et al. (2012) Nitrogen addition shapes soil phosphorus availability in two reforested tropical forests in Southern China. Biotropica 44: 302–311
39. Mirmanto E, Proctor J, Green J, Nagy L (1999) Effects of nitrogen and phosphorus fertilization in a lowland evergreen rainforest. Philosophical Transactions of the Royal Society 354: 1825–1829
40. Mo J, Brown S, Xue J, Fang Y, Li Z (2006) Response of litter decomposition to simulated N deposition in disturbed rehabilitated and mature forests in subtropical. China Plant and Soil 282: 135–151
41. Mo J, Li D, Gundersen P (2008) Seedling growth response of two tropical tree species to nitrogen deposition in southern China. European Journal of Forest Research 127: 275–283
42. Mo J, Zhang W, Zhu W, Fang Y, Li D, Zhao P (2007) Response of soil respiration to simulated N deposition in a disturbed and a rehabilitated tropical forest in southern China. Plant and Soil 296: 125–135
43. Mo J, Zhang WEI, Zhu W, Gundersen P, Fang Y, Li D, et al (2008) Nitrogen addition reduces soil respiration in a mature tropical forest in southern China. Global Change Biology 14: 403–412
44. Nomura N, Kikuzawa K (2003) Productive phenology of tropical montane forests: fertilization experiments along a moisture gradient. Ecological Research 18: 573–586
45. Ostertag R (2001) Effects of nitrogen and phosphorus availability on fine-root dynamics in Hawaiian montane forests. Ecology 82: 485–499
46. Ostertag R. (2010) Foliar nitrogen and phosphorus accumulation responses after fertilization: an example from nutrient-limited Hawaiian forests. Plant and Soil 334 85–98.
47. Priess JA, Fölster H (2001) Microbial properties and soil respiration in submontane forests of Venezuelan Guyana: characteristics and response to fertilizer treatments. Soil Biology and Biochemistry 33: 503–509
48. Raich JW, Russell AE, Crews TE, Farrington H, Vitousek PM (1996) Both nitrogen and phosphorus limit plant production on young Hawaiian lava flows. Biogeochemistry 32: 1–14
49. Reed SC, Cleveland CC, Townsed AR (2007) Controls over leaf litter and soil nitrogen fixaton in two lowland tropical rain forests. Biotropica 39: 585–592
50. Reed SC, Vitousek PM, Cleveland CC (2011) Are patterns in nutrient limitation belowground consistent with those aboveground: results from a 4 million year chronosequence. Biogeochemistry 106: 323–336
51. Solis E, Campo J (2004) Soil N and P dynamics in two secondary tropical dry forests after fertilization. Forest Ecology and Management 195: 409–418
52. Steudler PA, Garcia-Montiel DC, Piccolo MC, Neill C, Melillo JM, Feigl BJ, et al (2002) Trace gas responses of tropical forest and pasture soils to N and P fertilization. Global Biogeochemical Cycles 16: GB001394
53. Tanner EVJ, Kapos V, Franco W (1992) Nitrogen and phosphorus fertilization effects on venezuelan montane forest trunk growth and litterfall. Ecology 73: 78–86
54. Tanner EVJ, Kapos V, Freskos S, Healey JR, Theobald AM (1990) Nitrogen and phosphorus fertilization of Jamaican montane forest trees. Journal of Tropical Ecology 6: 231–238
55. Turner BL, Yavitt JB, Harms KE. Seasonal Changes and Treatment Effects on Soil Inorganic Nutrients Following a Decade of Fertilizer Addition in a Lowland Tropical Forest. Soil Sci. Soc. Am. J. 2013; 77:1357–1369.
56. Vitousek PM, Farrington H (1997) Nutrient limitation and soil development: Experimental test of a biogeochemical theory. Biogeochemistry 37: 63–75
57. Vitousek PM, Walker LR, Whiteaker LD, Matson PA (1993) Nutrient limitations to plant growth during primary succession in Hawaii Volcanoes National Park. Biogeochemistry 23: 197–215
58. Wright SJ, Yavitt JB, Wurzburger N, Turner BL, Tanner EVJ, Sayer EJ, et al. (2011) Potassium, phosphorus, or nitrogen limit root allocation, tree growth, or litter production in a lowland tropical forest. Ecology. 92: 1616–1625
59. Wu J, Ji C, Liu W, Fan H, Huang G, Wan S, Yuan Y (2013) Asynchronous responses of soil microbial community and understory plant community to simulated nitrogen deposition in a subtropical forest. Ecology and Evolution 3: 3895–3905
60. Wullaert H, Homeier J, Valarezo C, Wilcke W (2010) Response of the N and P cycles of an old-growth montane forest in Ecuador to experimental low-level N and P amendments. Forest Ecology and Management 260: 1434–1445
61. Xu G, Mo J, Fu S, Gundersen P, Zhou G, Xue J (2007) Response of soil fauna to simulated nitrogen deposition: A nursery experiment in subtropical. China Journal of Environmental Sciences-China 19: 603–609
62. Zhang W, Mo J, Yu G, Fang Y, Li D, Lu X, et al (2008) Emissions of nitrous oxide from three tropical forests in Southern China in response to simulated nitrogen deposition. Plant and Soil 306: 221–236
63. Zhang W, Mo J, Zhou G, Gundersen P, Fang Y, Lu X, et al (2008b) Methane uptake responses to nitrogen deposition in three tropical forests in southern. China Journal in Geophysical Research 113: D11116
64. Zhu F, Yoh M, Gilliam FS, Lu X, Mo J (2013) Nutrient Limitation in Three Lowland Tropical Forests in Southern China Receiving High Nitrogen Deposition: Insights from Fine Root Responses to Nutrient Additions. PLoS ONE 8: e82661. doi:10.1371/journal.pone.0082661
